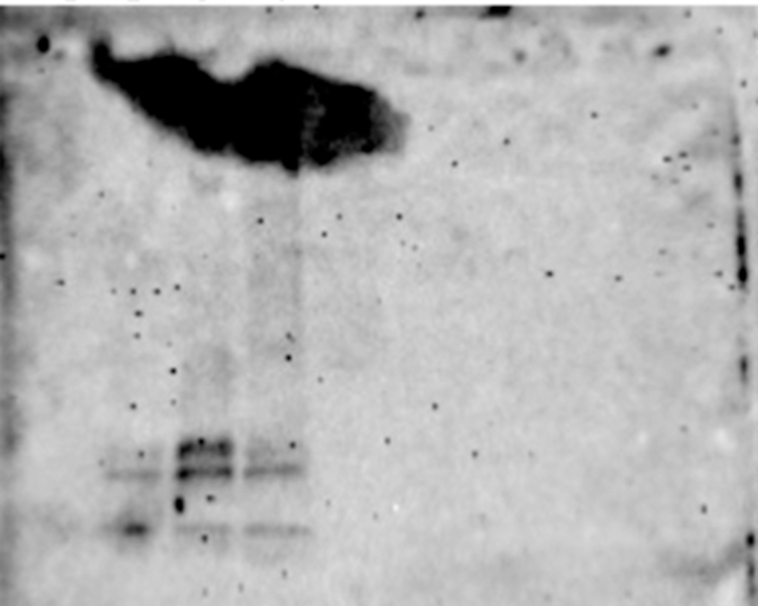# Chimeric hepatitis B surface antigen virus-like particles expressing the strep A epitope p*17 elicit a humoral immune response in mice

**DOI:** 10.1016/j.heliyon.2024.e30606

**Published:** 2024-05-04

**Authors:** Leanne Dooley, Tarek Ahmad, Victoria Ozberk, Manisha Pandey, Michael Good, Michael Kotiw

**Affiliations:** a School of Health and Medical Sciences, University of Southern Queensland, Toowoomba, Queensland, Australia; b Institute for Life Sciences and the Environment, University of Southern Queensland, Toowoomba, Queensland, Australia; c Institute for Glycomics, Griffith University, Gold Coast, Queensland, Australia

## Abstract

To optimize immunogenicity, bacterial epitopes in putative vaccine constructs can be presented to immune cells as multiple repeated structures on a defined nanoparticle. Virus-like particles (VLPs) are viral capsid proteins that self-assemble to form compact and highly ordered nanoparticles that are within the optimal size range for uptake by dendritic cells. VLPs mimic the live virus in size and form but contain no viral genetic material, are therefore noninfective and are the basis of safe and effective vaccines against hepatitis B virus (HBV) and human papillomavirus (HPV). Due to their particulate nature, molecular stability, and expression of high density and repetitive antigen displays, recombinant cell culture-derived VLPs are ideal platforms for the delivery of small molecules, including bacterial epitopes. We developed a putative vaccine by expressing a minimal epitope from the bacterium *Streptococcus pyogenes* (Strep A) on the surface of a recombinant VLP comprising multiple copies of HBV small envelope protein (HBsAg-S). Strep A is responsible for a wide spectrum of human infections and postinfectious diseases that disproportionately affect children and young adults living in resource-poor communities. No vaccine is currently available to offer sufficiently broad protection from the numerous and diverse strains of Strep A endemic in these at-risk populations. The Strep A antigen targeted by our vaccine construct is p*17, a cryptic epitope from a highly conserved region of the Strep A M-protein with demonstrated enhanced immunogenicity and broad protective potential against Strep A. To ensure surface expression and optimal immunogenicity, we expressed p*17 within the immunodominant “a” determinant of HBsAg-S. The recombinant VLPs (VLP-p*17) expressed in HEK293T cells spontaneously formed 22 nm particles and induced the production of high titers of p*17-specific IgG in BALB/c mice immunized with three 0.5 μg doses of VLP-p*17 formulated with adjuvant.

## 1 Introduction

Despite universal susceptibility to penicillin, *Streptococcus pyogenes* (Strep A) claims an estimated 500 000 lives globally every year [1,2]. The most common causes of Strep A-related deaths are severe invasive infections and rheumatic heart disease (RHD), an autoimmune sequela of superficial Strep A infections [3,4]. A disproportionate share of the Strep A-associated disease burden is carried by children and young adults in developing countries and in indigenous communities within developed countries, including Australia, Canada, New Zealand, and the United States of America [2,5]. Vaccination is currently widely considered the best means of prophylaxis against Strep A, and despite decades of research, no vaccine offering adequate protection to at-risk populations is currently available [6]. A major challenge to Strep A vaccine development has been the vast serotypical and epidemiological diversity of the organism. Hence, most Strep A vaccine candidates currently under development target conserved epitopes expressed by all pathogenic strains of the bacterium. Many contemporary Strep A vaccine candidates target epitopes derived from p145, a 20 amino acid peptide within a highly conserved C-terminal region of the Strep A M protein: a dimeric alpha-helical protein that covers the surface of the bacterium and protects it from complement-mediated phagocytosis [7] (Fig. 1). The highly conserved nature of p145 and observations that anti-p145 antibodies develop naturally in response to Strep A infection have made p145 an attractive target for a broad-spectrum Strep A vaccine [8]. Several vaccine constructs utilizing a range of different platforms to deliver various derivatives of p145 have been developed by Australian researchers. While these constructs demonstrated immunogenicity, multiple immunizations were needed for adequate protection in mouse models of Strep A infection, and only one construct, a p145 derivative conjugated to Diphtheria toxoid (MJ8VAX), has progressed to human trials (reviewed in Ref. [6]).

**Fig. 1:**
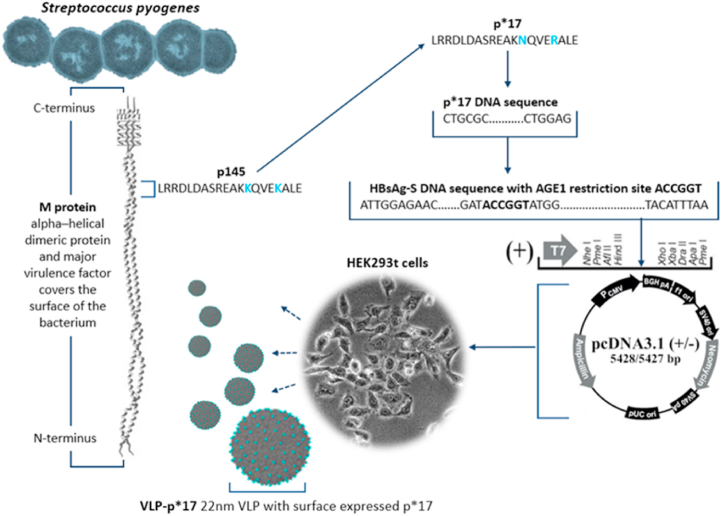
Design and synthesis of VLP-p*17 (amino acid substitutions in p145 to create p*17 are shown in blue). The p*17 DNA sequence was incorporated into the HBsAg-S DNA sequence at an engineered *AGE1* restriction site. The HBsAg-S-p*17 sequence was then inserted into the pcDNA3.1 expression vector and expressed in HEK293T cells, which subsequently secreted recombinant VLP-p*17. (For interpretation of the references to colour in this figure legend, the reader is referred to the Web version of this article.)

To increase the immunogenicity of p145, Nordstrom et al. (2017) designed 18 peptides through a process of substituting one amino acid at a time in the aa sequence of p145 and assessed the immunogenicity of the new peptides in outbred mice. One of these peptides, p*17 (p145 with two amino acid substitutions, as shown in Fig. 1), induced more than 10 000-fold greater protection following Strep A challenge when conjugated to diphtheria toxoid (DT) and formulated with alum than a p145-DT conjugate formulated with alum. Furthermore, three immunizations with p145-DT/Alum were needed to induce complete protection from Strep A, while only a single immunization with p*17-DT/Alum was needed [9].

To optimize immunogenicity, bacterial epitopes in putative vaccine constructs can be presented as multiple repeated structures on a defined nanoparticle such as a virus-like particle (VLP) [10]. VLPs comprising multiple monomers of hepatitis B virus small envelope protein (HBsAg-S) form the basis of the currently used second generation vaccines against hepatitis B, Energix-B (GlaxoSmithKline) and Recombivax HB® (Merck & Co. Inc) and the anti-malarial vaccine RTS,S (Mosquirix®) [[11], [12], [13]]. Recombinant-derived HBsAg-S molecules spontaneously polymerize to form 22 nm octahedral particles, each comprising approximately 100 HBsAg-S monomers [14]. Due to their particulate nature, molecular stability, high density and repetitive display of epitopes, HBsAg-S VLPs are ideal platforms for the delivery of small molecules, including bacterial epitopes [10,11,15,16]. The HBsAg-S amino acid sequence contains a highly conformational, hydrophilic domain between positions 122 and 150 referred to as the “a” determinant [17,18]. The “a” determinant is expressed on the surface of the VLP and contains epitopes for the induction of a protective humoral immune response [19].

We hypothesized that insertion of the minimal Strep A epitope p*17 into the “a” determinant of HBsAg-S would preserve the immunogenicity of p*17 and would not inhibit spontaneous polymerization of the HBsAg-S monomers. We also hypothesized that combining the enhanced immunogenicity of p*17 with the natural immunogenicity of HBsAg-S VLPs for epitope delivery would produce a putative vaccine capable of triggering the production of high-titer p*17-specific IgG in mice immunized with VLP-p*17 formulated with adjuvant. Therefore, the aim of this study was to utilize recombinant DNA technology to express p*17 on the surface of HBsAg-S VLPs, to semi-purify cell culture-derived VLP-p*17 and to evaluate its immunogenicity in mice.

## 2 Materials and methods

Utilizing recombinant DNA technology, we designed and produced a chimeric HBsAg VLP with surface-expressed p*17 (Fig. 1) and evaluated the p*17-specific humoral response in mice vaccinated with three 0.5 μg doses of the putative Strep A vaccine, VLP-p*17.

### 2.1 DNA expression vector

The DNA sequence for the recombinant HBsAg-S-p*17 construct (VLP-p*17) was designed with the p*17 sequence inserted at an engineered *AGE1* restriction site in the cDNA of HBsAg-S (between amino acids 127 and 128) to direct expression of p*17 in the “a” determinant region of HBsAg-S [15,20]. The VLP-p*17 gene was codon optimized for expression in mammalian cells, synthesized and cloned and inserted into the *Hin*dIII/*Xho*I cloning site of pcDNA3.1(+) by GenScript (Hong Kong) Limited, and purchased as 300 μg of bespoke transfection grade plasmid in 300 μl buffer.

### 2.2 Anti-p*17 antibodies

Sera were raised in BALB/c mice immunized with three doses of p*17-DT/Alum as part of a study by Nordstrom et al. (2017). The sera were tested by ELISA and were shown to contain p*17-specific IgG antibodies (titers 10^5^) [9]. The sera were used in this study at a 1:32 000 dilution as a source of anti-p*17 antibodies for Western blotting.

### 2.3 Synthesis of cell culture-derived VLP-p*17

Three 2 ml vials of HEK293T (ATCC® CRL-3216™) cells were resuscitated from storage in 10 % dimethyl sulfoxide at −80 °C by gentle thawing (in a closed hand). The DMSO was removed by discarding the supernatant after centrifugation for 8 min at 125 g. Cells were immediately resuspended in 2 ml Dulbeco's Modified Eagle's Medium-High glucose (DMEM Sigma® Life Science) with 10 % fetal bovine serum (FBS, Australia origin Sigma‒Aldrich), 1 % l-glutamine 200 mM (GlutaMAX™ 100X Gibco), and 2 % penicillin 5000 U/ml-streptomycin 5 mg/ml (Sigma‒Aldrich). The three vials of resuspended cells were then immediately transferred to three 250 ml CELLSTAR® cell culture flasks with filter caps (Greiner Bio-One, Kremsmünster, Austria), each containing 20 ml of cell culture media as described above. Flasks were incubated at 37 °C with 5 % CO_2,_ and cells were passaged on alternate days for 10 days, after which time they were estimated to be 70–80 % confluent. HEK293T cells were transfected using FuGENE® HD Transfection Reagent (Promega) following the manufacturer's instructions with a 3:1 ratio of FuGENE®HD (900 μl) to DNA (300 μl pcDNA3.1(+)-HBsAg-S-p*17) in 1.8 ml of FBS-free DMEM. The transfection mixture was vortexed briefly and incubated at room temperature for 15 min, after which 1 ml was added to the HEK293T cells in each of the three flasks. The flasks were then gently mixed in a shaking incubator at 37 °C for 10 min. The transfected HE293T cells were incubated at 37 °C with 5 % CO_2_ for five days with an additional 10 ml of prewarmed FBS-free DMEM added to each flask daily. The supernatant from each cell culture flask was harvested on day six post transfection and analyzed by SDS‒PAGE using Mini-PROTEAN® TGX™ Precast Gels (Bio-Rad) following the manufacturer's instructions. A 300 μl sample of crude supernatant from each flask was also analyzed for HBsAg concentration using an automated chemiluminescent microparticle assay (Abbott ARCHITECT) [21].

### 2.4 Partial purification and quantitation of culture-derived VLP-p*17

50 ml of crude cell culture supernatant containing the recombinant VLPs was removed from each flask and centrifuged at 2400 g (Heraeus Multifuge Thermo Scientific) for 10 min, to remove cellular debris, and then transferred to six 38.5 ml Open-Top Thinwall Ultra-Clear™ (Beckman Coulter) centrifuge tubes. The supernatant in each centrifuge tube was then underlaid with 5 ml 20 % sucrose in STE buffer (100 mM NaCl, 10 mM Tris, pH 8, 1 mM EDTA), and the VLPs were pelleted by ultracentrifugation at 25 000 rpm (Beckman Optima XPN-100) for 16 h at 4 °C. The supernatant was discarded, and the pelleted VLPs in each of the six tubes were resuspended in 200 μl STE buffer with three 30 s bursts of low-speed sonication in a sonicator-water bath (GT Sonic) at 4 °C. Partially purified and resuspended VLP samples were further diluted 1:2 in STE buffer, and aliquots were again analyzed for HBsAg concentration, as described in Section 2.3. The remainder of each sample was then stored in 200 μl aliquots at −80 ^°^C until needed.

### 2.5 Transmission electron microscopy of partially purified VLP-p*17

Three 200 μl samples of partially purified VLP-p*17 were analyzed by transmission electron microscopy (TEM) following a standard negative stain protocol at the Centre for Microscopy and Microanalysis (CMM), University of Queensland, Australia. Four microliters of each sample were placed onto Formvar-coated copper grids for 1 min. Excess sample was removed with damp filter paper, after which 1 % uranyl acetate was added to each grid for 1 min and then completely removed with filter paper. The grids were allowed to dry before being viewed in a Hitachi HT7700 transmission electron microscope at 100 kV.

### 2.6 Detection of p*17 expression by Western blot

Three 200 μl aliquots of partially purified VLP-p*17 in STE buffer were retrieved from frozen storage, thawed at room temperature and analyzed for p*17 expression by Western blot utilizing polyclonal murine anti-p*17-DT sera diluted 1:32 000 in Pierce® Fast Western Antibody Diluent (Thermo Scientific). Proteins were separated by SDS‒PAGE (200 v for 30 min), transferred to a nitrocellulose membrane, and blotted using a Pierce® Fast Western blot Kit with enhanced chemiluminescence (ECL) Substrate (Thermo Scientific) following the manufacturer's instructions.

### 2.7 Formulation of VLP-p*17 with adjuvant

Aliquots of VLP-p*17 in STE buffer were retrieved from frozen storage, thawed at room temperature, and pooled into two lots of equal volume, each of which was then diluted in 30 ml sterile PBS and stored overnight at 4 °C. The VLPs in PBS were pelleted again by overnight ultracentrifugation at 25 000 rpm and 4 °C. The supernatant was discarded, and the VLPs were resuspended in 400 μl sterile PBS to replace the STE buffer with a physiological buffer in preparation for immunization of mice. Fifty microliters were removed and added to 250 μl of PBS for final HBsAg quantitation. The result was 8200 IU/ml, which according to the conversion described by Deguchi et al. (2004), where 0.05 IU/ml HBsAg approximates 0.2 ng/ml HBsAg, equates to approximately 33 μg/ml HBsAg [22]. Immediately prior to formulation with adjuvant, VLP-p*17 in PBS was filtered using a 0.22 μm Millex-GP Syringe Filter Unit (Merck Millipore, Ltd., Ireland). For each single vaccine dose, 5 μl of alum (40 mg/ml aluminum hydroxide, Vaxine Pty. Ltd., Adelaide, Australia) was added to 15 μl of VLP-p*17 and 15 μl of sterile PBS for a total volume of 35 μl per dose per mouse.

### 2.8 Mouse immunization

All protocols were approved by the Griffith University Animal Ethics Committee and ratified by the University of Southern Queensland Animal Ethics Committee in compliance with Australian National Health and Medical Research guidelines. The study involved four groups of 4–6-week-old BALB/c mice (Animal Resource Centre Australia) (n = 5 per group). Mice were acclimatized for 7 days prior to immunization. On Day 0, one group of mice was inoculated i.m. in the hind thigh with 0.5 μg VLP-p*17 formulated with alum in sterile PBS as described above. The dosage of 0.5 μg for VLP-p*17 was chosen in keeping with the 0.25–0.5 μg doses used in animal trials of other VLP-based vaccines [11,15,20]. A second group was inoculated with 5 μl of alum in 30 μl of sterile PBS as an adjuvant control, and the third group was inoculated with 35 μl of sterile PBS alone as a negative control. As a positive control to validate ELISA results, a fourth group was inoculated with 25 μg p*17-DT formulated with 25 μl of alum. The dosage of 25 μg for p*17-DT was chosen in keeping with that used by Nordstrom et al. (2017). Each mouse received a booster dose of the same formulation as for the original inoculation on Days 21 and 28. Blood samples (maximum 200 μl) were collected from the submandibular vein of each mouse one day prior to boosting and one week after the final boost. The blood was allowed to clot at 4 °C for at least 4 h, and serum was separated after centrifugation at 1000×*g* for 10 min. Sera were stored at −80 °C.

### 2.9 ELISA

Standard ELISA was used to determine murine p*17-specific IgG antibody titers. Ninety-six-well microtiter plates (Nunc, Denmark) were coated with 5 μl (10 μg/ml) p*17 peptide (ChinaPeptides) in 10 ml carbonate buffer (pH 9.6) (100 μl/well) for 90 min at 37 °C. The plates were blocked overnight at 4 °C with 150 μl/well 5 % skim milk in PBS with 0.05 % Tween 20 and then washed twice with PBS/0.05 % Tween 20. Murine serum samples were diluted 1/100 in 0.5 % skim milk and then added in duplicate across the plate with doubling dilutions down the plate (100 μl/well). The plates were incubated for 90 min at 37 °C and then washed four times with PBS/0.05 % Tween 20. Goat anti-mouse IgG (H + L)-HRP conjugate (Bio-Rad, Australia) diluted 1/3000 in 0.5 % skim milk was added (100 μl/well), and the plates were once again incubated for 90 min at 37 °C and then washed four times with PBS/0.05 % Tween 20. TMB substrate solution (Thermo Scientific) was prepared according to the manufacturer's instructions, and 100 μl was added to each well. The plates were incubated at room temperature in the dark for 10 min, after which time the reactions were stopped with 100 μl of 2 M sulfuric acid, and the absorbance for each plate was read at 450 nm using a Varioskan LUX Multimode Microplate Reader (Thermo Fisher) with SkanIt Software 6.0.2.3 for Microplate Readers. Titers were determined using the mean absorbance of the negative control group (PBS alone) plus three standard deviations as the cutoff.

## 3 Results

### 3.1 Secretion competence of cell culture-derived VLP-p*17

**Fig. 2:**
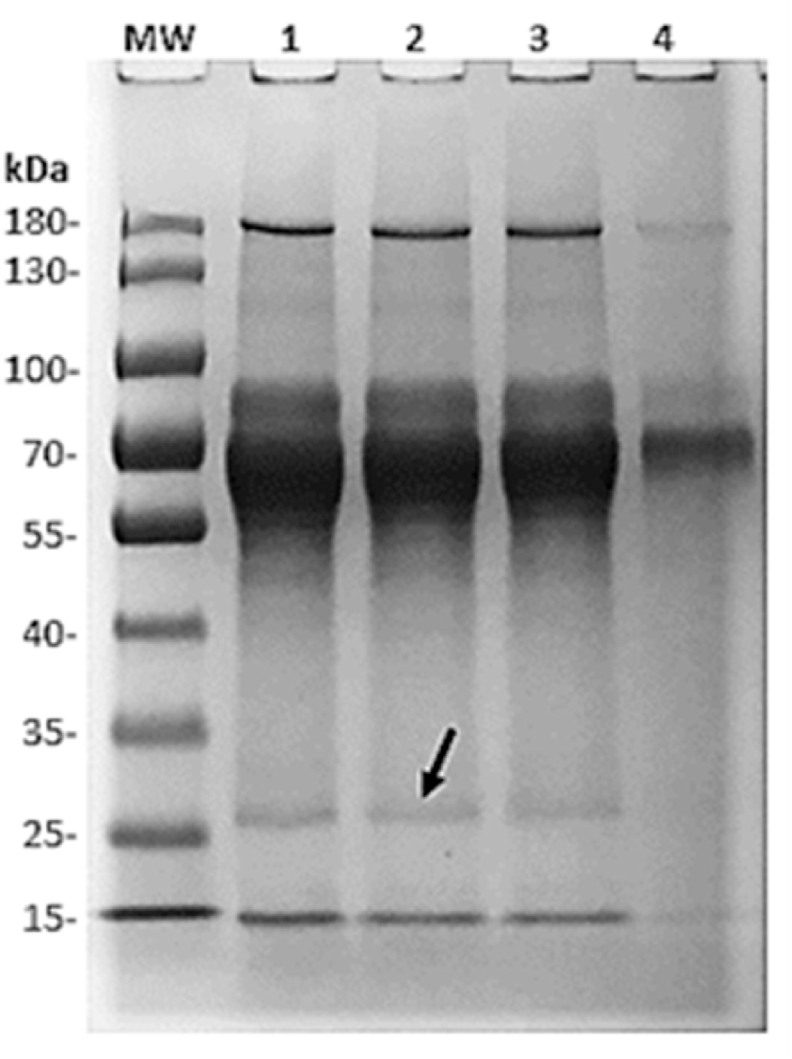
SDS‒PAGE of crude supernatant from three cell culture flasks of HEK293T cells (Lanes 1, 2 and 3) six days post transfection showing a band at approximately 29 kDa (arrow). MW shows a prestained 10–180 kDa protein marker (Servicebio®), and Lane 4 contained crude pre-transfection cell culture supernatant as a negative control. Imperial™ Protein Stain (Thermo Scientific). (Uncropped SDS-PAGE image provided as suplementary material).

The molecular weight (MW) of VLP-p*17 (HBsAg-S + p*17) was calculated at 29.198 kDa (Bioinformatics.org). The presence of bands at approximately 29 kDa after SDS‒PAGE of the post transfection cell culture supernatant suggested that the recombinant VLPs (VLP-p*17) were secretion competent (Fig. 2). This was confirmed by the results of the HBsAg assay shown in Table 1. The mean HBsAg concentration of the crude post transfection supernatant samples was 24.2 IU/ml. According to the conversion described by Deguchi et al. (2004), this result approximates 96.8 ng/ml HBsAg [22].

**Table 1:** HBsAg quantitation in three HEK293T cell culture supernatant samples post transfection with VLP-p*17 pcDNA3.1(+) and post 20 % sucrose gradient ultracentrifugation.

Sample		HBsAg IU/ml	HBsAg ng/ml^a^	VLP/ml^a^
Crude HEK293T cell culture supernatant post transfection	1	20.26	81.04	1.6 × 10^10^
	2	29.93	119.7	2.4 × 10^10^
	3	22.41	89.6	1.7 × 10^10^
Resuspended pelleted VLPs post ultracentrifugation through 20 % sucrose	1	553	2212	4.4 × 10^11^
	2	1425	5701	1.1 × 10^12^
	3	628	2515	5.0 × 10^11^

a 0.05 IU/ml HBsAg ∼ 0.2 ng/ml HBsAg and 0.1 ng/ml HBsAg ∼ 2 x 10^7^VLPs [22].

### 3.2 Characterization of cell culture-derived VLP-p*17

**Fig. 3:**
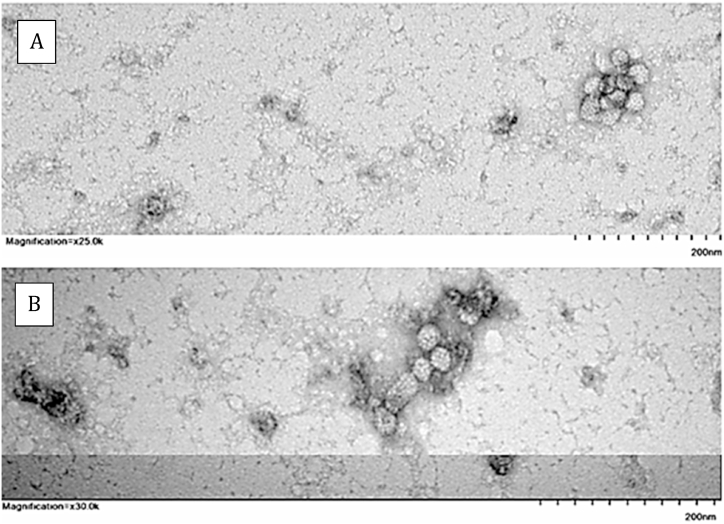
Transmission electron micrograph showing clusters of virus-like particles with visible capsomeres in partially purified cell culture supernatant. Using the scale provided, each particle appears to be approximately 22 nm in diameter. Panel A shows a cluster of VLPs at 25.0k magnification and panel B shows another cluster of VLPs at 30.0k magification. Image by Kathryn Green, CMM.

To demonstrate particle formation, crude supernatant was concentrated and partially purified by overnight ultracentrifugation through a 20 % sucrose cushion as described above. The pelleted contents were resuspended in 200 μl STE buffer, analyzed for HBsAg (Table 1) and visualized using transmission electron microscopy. The TEM images (Fig. 3) confirmed the presence of homologous particles approximately 22 nm in diameter with visible viral capsomeres. These images are consistent with our previously published TEM images of recombinant HBsAg VLPs [15]. The mean HBsAg concentration of the concentrated and partially purified samples was 868.7 IU/ml, which converts to approximately 3475 ng/ml. According to Deguchi et al. (2004), 0.1 ng/ml HBsAg approximately equals 2 × 10^7^ VLPs. Using this calculation, the mean concentration of recombinant HBsAg VLPs in the concentrated and partially purified samples was approximately 6.9 × 10^11^/ml [22].

### 3.3 Detection of p*17 expression by Western blot

**Fig. 4:**
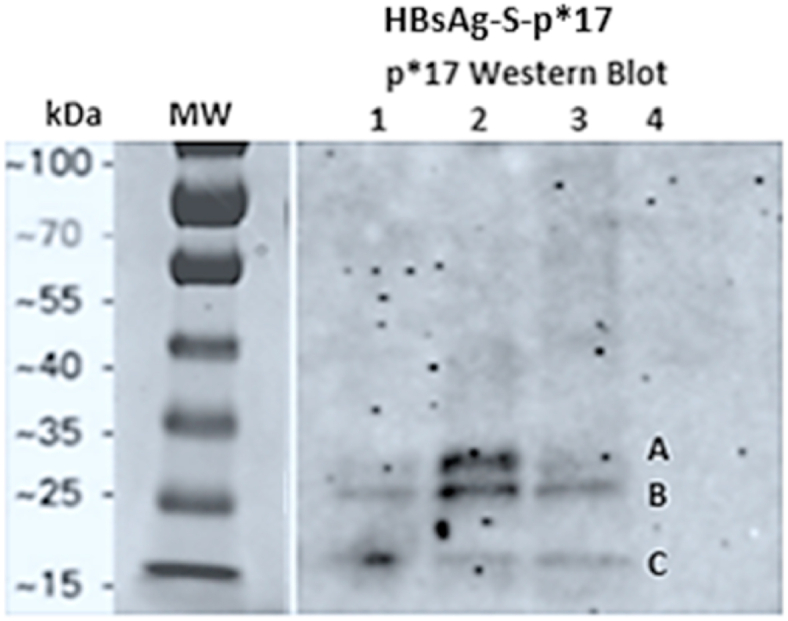
Western blot utilizing anti-p*17-DT sera. The panel on the left illustrates the migration of the MW marker (Servicebio®) during SDS‒PAGE for comparison with the Western blot on the right, which illustrates the presence of glycosylated (A) and nonglycosylated (B) HBsAg-S-P*17 monomers and truncated or degraded monomers (C) in resuspended pelleted VLPs after ultracentrifugation through a 20 % sucrose cushion (diluted 1:5) in Lanes 1, 2 and 3. The darker bands in Lane 2 are consistent with the significantly higher concentration of VLP measured in sample 2, as shown in Table 1. Lane 4 contained HBsAg (MyBioSource) as a control for nonspecific binding of anti-p*17-DT to the HBsAg-S component of VLP-p*17. (Uncropped Western blot image provided as sumplementary material).

The expression of p*17 in the recombinant VLPs was confirmed by Western blot analysis of concentrated and partially purified post transfection cell culture supernatant using sera from mice previously immunized with p*17-DT/Alum. The anti-p*17-DT antibodies recognized VLP-p*17, confirming the expression of p*17 in the recombinant VLPs (Fig. 4). Wild-type HBsAg-S VLPs comprise a mixture of nonglycosylated (P24) and glycosylated (GP27) HBsAg monomers [23]. This mix of glycosylated and nonglycosylated forms is reflected in the presence of bands at 31 kDa and 29 kDa. The band at 17 kDa most likely indicates the presence of truncated or degraded monomers that still express p*17.

### 3.4 ELISA

**Fig. 5:**
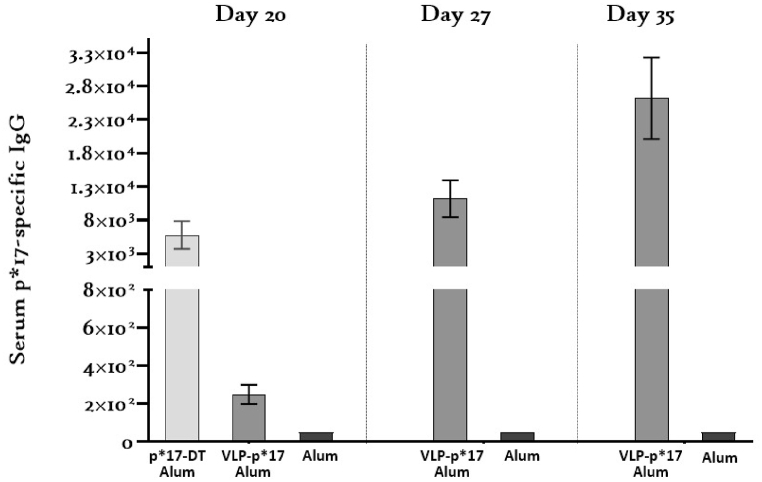
p*17-specific IgG titers in sera collected on Days 20, 27 and 35 from mice immunized with VLP-p*17 formulated with alum in sterile PBS or with alum only in sterile PBS or with p*17-DT (positive control).

p*17-specific IgG was demonstrated in the sera of all mice immunized with VLP-p*17 or p*17-DT formulated with alum in sterile PBS and in no mice inoculated with alum in PBS or with PBS only. While the p*17-specific IgG titers were low (range 2 × 10^2^ - 8 × 10^2^) after the initial inoculation with 0.5 μg VLP-p*17, they increased at least 32-fold after the first booster dose to greater than 1.28 × 10^4^ (range 1.28 × 10^4^–8 × 10^4^) after the second booster dose (Fig. 5).

## 4 Discussion

In this study, we designed and produced a novel Strep A vaccine candidate utilizing a recombinant hepatitis B small envelope protein (HBsAg-S) virus-like particle (VLP) as the delivery platform for the cryptic highly conserved and highly immunogenic Strep A M protein epitope, p*17. This putative vaccine, VLP-p*17, induced the production of measurable p*17-specific IgG in the sera of mice after a single 0.5 μg dose formulated with the adjuvant alum (mean titer 3.6 × 10^2^). The antibody titers rose approximately 36-fold (mean titer 1.3 × 10^4^) after a second 0.5 μg dose and then doubled again (mean titer 3.3 × 10^4^) after a third 0.5 μg dose (Fig. 5). The data presented in Fig. 5 for the positive control group, immunized with 25 μg p*17-DT formulated with 25 μl of alum, correlate with the high p*17-specific titers demonstrated by Nordstrӧm et al. (2017). This group, with the higher inoculum dose, was included in the study only as a control to validate ELISA results in the event that mice immunized with VLP-p*17 failed to produce measurable titers of p*17-specific IgG, which was not the case. However, it was interesting to note that while there was a 50-fold difference in the antigen dose, the difference in the serum p*17-specific IgG tires between the test group (mean 2.5 × 10^2^) and the positive control group (mean 6.4 × 10^3^) was only 25-fold. This could suggest enhanced immunogenicity of VLP-p*17 over p*17-DT, although a dose-matched trial would be needed to demonstrate this conclusively.

Culture-derived VLPs are naturally immunogenic due to their morphological similarity to the parent virus, which extends to both the lipid and protein content of the highly repetitive viral envelope structure. *In vivo*, because of their size and particulate nature, culture-derived HBsAg VLPs are readily taken up by DCs and are processed via the same immune pathways as the parent virus [24]. In addition to their value as antiviral vaccines, VLPs are highly stable molecules that have demonstrated tolerance to a range of bioconjugation, labeling, and polymerization techniques, making them suitable delivery platforms for a wide spectrum of small molecules, including bacterial epitopes [14]. Furthermore, VLPs can be expressed in and secreted by a range of protein expression systems, making them relatively easy and inexpensive to produce [25]. The secretion competence of the recombinant VLP-p*17 expressed in HEK293T cells was confirmed by HBsAg quantitation of the crude and partially purified cell culture supernatant (Table 1). To facilitate surface expression and ensure optimal immunogenicity of the Strep A epitope, p*17 was expressed within the immunodominant “a” determinant region of HBsAg-S. The inherent immunogenicity of HBsAg-S stems from the spontaneous assembly of individual monomers into highly organized 22 nm particles [23]. It was therefore important to determine whether insertion of the foreign peptide sequence LRRDLDASREAKNQVERALE into the “a” determinant of HBsAg-S enabled particle formation. The TEM images (Fig. 3) show homologous particles approximately 22 nm in diameter with visible capsomeres, confirming that insertion of the 20 mer foreign peptide into the “a” determinant of HBsAg-S did not prevent particle formation. This approach differs from that used in the development of HBsAg-S VLP-based antimalarial vaccines, which comprise a peptide from the circumsporozoite protein (CSP) of *Plasmodium falciparum* fused to the N-terminus of HBsAg-S. While this configuration also ensures surface expression of the target antigen, it interferes with particle formation. In the RTS,S anti-malaria vaccine, the interference was minimized by incorporation of wild-type HBsAg-S such that each VLP contained a somewhat random mix of both wild-type HBsAg-S and CSP-HBsAg. This diluted the expression of CSP and thereby reduced the CSP-specific antibody response in vaccinated mice [11]. Rather than fusing or conjugating a Strep A epitope to the N-terminus of HBsAg-S, this project involved the use of recombinant DNA technology to clone the bacterial epitope into a more central yet still surface-expressed region of HBsAg-S without compromising particle formation or immunogenicity.

Immunization of mice with 0.5 μg VLP-p*17 formulated with alum induced measurable titers of p*17-specific IgG after a single dose (mean 3.6 × 10^2^) that increased to greater than 1.28 × 10^4^ (mean 3.3 × 10^4^) after two 0.5 μg booster doses. Higher initial doses of VLP-p*17, such as those used for the p*17-DT positive control group, could induce sufficiently high IgG titers without the need for boosting.

This putative vaccine, VLP-p* 17, has features that make it an attractive construct for a commercially produced vaccine against Strep A: it is a single recombinant protein that spontaneously assembles into stable particles that are of optimal size for recognition and uptake by human immune cells; it presents repetitive displays of the highly immunogenic Strep A epitope, p*17, on the surface of homologous nanoparticles; it can be easily and consistently produced in yeast or mammalian cells; and it is based on the HBsAg-S VLP, which is already licensed as a human vaccine with associated large-scale quality-controlled manufacturing processes already in place.

While several VLP-based antiviral and antimalarial vaccines have been approved for human use, to our knowledge, no VLP-based antibacterial vaccine has progressed to clinical trials, further highlighting the novelty of VLP-p*17 [12].

## 5 Conclusion

In conclusion, we developed a recombinant HBsAg-S VLP expressing the minimal Strep A M protein epitope, p*17. This construct, VLP-p*17, combines the natural immunogenicity of VLPs with a cryptic highly conserved Strep A epitope that has demonstrated enhanced immunogenicity and efficacy when conjugated with DT. VLP-p*17 induced the production of consistently high titers of p*17-specific IgG after three 0.5 μg doses. Furthermore, the parent VLP utilized in this construct, HBsAg-S, is already approved for use in human vaccines, which may remove some obstacles to future human trials of VLP-p*17. A limitation of this study is the partial purification of VLP-p*17. Further purification could have resulted in significant loss of VLPs and was not essential for this proof-of-principle study. However, we acknowledge that impurities in the vaccine formulation could have had an adjuvant effect, or a suppressive effect, on the humoral response to immunization, and further studies with more highly purified VLPs should follow. Other limitations of the study are the absence of functional assays of murine serum p*17-specific IgG and a dose-matched study comparing the antibody responses in mice immunized with VLP-p*17 and mice immunized with p*17-DT, which will be conducted as part of a future study.

## Ethics declarations

This study was conducted in accordance with the Australian Code for the Care and Use of Animals for Scientific Purposes 8th edition 2013, the Queensland Animal Care and Protection Act (2001), and the Griffith University Guidelines for Animal Care and Use in Teaching and Research.

This study was reviewed and approved by the Griffith University Animal Ethics Committee with the approval number GLY/07/21/AEC.

Informed consent was not needed for this study, which did not involve human participants.

## Funding

This research received no external funding.

## Data availability statement

Data included in the supplementary material.

## CRediT authorship contribution statement

**Leanne Dooley:** Writing – review & editing, Writing – original draft, Methodology, Investigation, Formal analysis, Data curation, Conceptualization. **Tarek Ahmad:** Methodology, Investigation, Data curation, Conceptualization. **Victoria Ozberk:** Methodology, Investigation. **Manisha Pandey:** Resources, Project administration, Methodology, Investigation, Conceptualization. **Michael Good:** Resources, Project administration, Funding acquisition. **Michael Kotiw:** Writing – review & editing, Supervision, Project administration, Funding acquisition, Conceptualization.

## Declaration of competing interest

The authors declare that they have no known competing financial interests or personal relationships that could have appeared to influence the work reported in this paper.

## Figures and Tables

**figs1:**
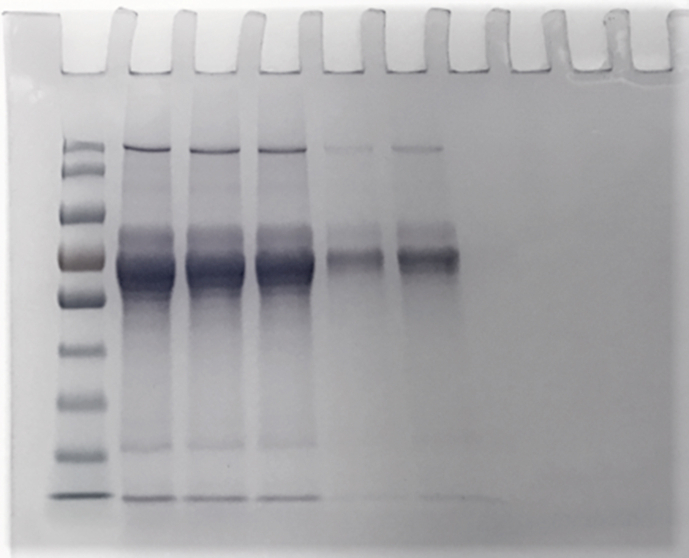


**figs2:**